# Association of *RERG* Expression with Female Survival Advantage in Malignant Pleural Mesothelioma

**DOI:** 10.3390/cancers13030565

**Published:** 2021-02-02

**Authors:** Assunta De Rienzo, Melissa H. Coleman, Beow Y. Yeap, David T. Severson, Benjamin Wadowski, Corinne E. Gustafson, Roderick V. Jensen, Lucian R. Chirieac, William G. Richards, Raphael Bueno

**Affiliations:** 1 Thoracic Surgery Oncology Laboratory and The International Mesothelioma Program, Division of Thoracic Surgery, Brigham and Women’s Hospital, Harvard Medical School, 75 Francis Street, Boston, MA 02115, USA; melissa.coleman@ucsf.edu (M.H.C.); dseverson@bwh.harvard.edu (D.T.S.); bwadowski@bwh.harvard.edu (B.W.); cgustafson1@bwh.harvard.edu (C.E.G.); wrichards@bwh.harvard.edu (W.G.R.); rbueno@bwh.harvard.edu (R.B.); 2 Department of Surgery, University of California San Francisco, 500 Parnassus Ave, MUW 405, Box 0118, San Francisco, CA 94143, USA; 3 Department of Medicine, Massachusetts General Hospital, Harvard Medical School, 55 Fruit Street, Boston, MA 02114, USA; BYEAP@mgh.harvard.edu; 4 Department of Biological Sciences, Virginia Tech, 970 Washington Street SW, Blacksburg, VA 24061, USA; rvjensen@vt.edu; 5 Department of Pathology, Brigham and Women’s Hospital, Harvard Medical School, 75 Francis Street, Boston, MA 02115, USA; lchirieac@bwh.harvard.edu

**Keywords:** malignant pleural mesothelioma, *RERG*, sex, estrogen, survival

## Abstract

**Simple Summary:**

Sex differences in tumor incidence and mortality have been documented for many different cancer types. In malignant pleural mesothelioma, a deadly disease, many studies have shown that women not only develop this cancer less frequently than men, but those who do are likely to live longer after surgery. These differences have been postulated to reflect circulating estrogen levels and tumor expression of estrogen receptors that may influence tumor progression. We identified high expression of the RAS like estrogen regulated growth inhibitor gene (*RERG*), to correlate with longer survival after surgery among women. Survival in men was not associated with *RERG* expression. Additionally, we found no association between survival and tumor expression of estrogen receptor genes. Additional studies are needed to elucidate any role *RERG* may play in mesothelioma, and whether estrogen may be involved.

**Abstract:**

Sex differences in incidence, prognosis, and treatment response have been described for many cancers. In malignant pleural mesothelioma (MPM), a lethal disease associated with asbestos exposure, men outnumber women 4 to 1, but women consistently live longer than men following surgery-based therapy. This study investigated whether tumor expression of genes associated with estrogen signaling could potentially explain observed survival differences. Two microarray datasets of MPM tumors were analyzed to discover estrogen-related genes associated with survival. A validation cohort of MPM tumors was selected to balance the numbers of men and women and control for competing prognostic influences. The RAS like estrogen regulated growth inhibitor (*RERG*) gene was identified as the most differentially-expressed estrogen-related gene in these tumors and predicted prognosis in discovery datasets. In the sex-matched validation cohort, low *RERG* expression was significantly associated with increased risk of death among women. No association between *RERG* expression and survival was found among men, and no relationship between estrogen receptor protein or gene expression and survival was found for either sex. Additional investigations are needed to elucidate the molecular mechanisms underlying this association and its sex specificity.

## 1. Introduction

Many cancers exhibit significant differences between men and women in terms of incidence, prognosis and mortality [1]. Malignant pleural mesothelioma (MPM) is a rare but highly lethal pleural-based malignancy associated with asbestos exposure. MPM exhibits a strong sex bias in terms of distribution (M:F = 4:1), likely due to occupational exposure and women with MPM have consistently demonstrated a significant survival advantage [2,3,4], even when controlling for other known prognostic factors including age, stage, and histologic subtype (ranging from epithelioid to sarcomatoid differentiation with increasingly poor prognosis) [5,6]. This survival advantage has been attributed to higher probability of occupational exposure to asbestos in men, whereas women generally have a non-occupational or secondhand exposure through spouses’ clothing, low-level environmental exposure, and other sources [5,6]. Alternatively, differences in molecular factors have also been considered. For example, some sex-related differences in typical MPM molecular features, such as mutation of *CDKN2A* and *TP53*, have already been described [7]. However, there is growing evidence suggesting that the female survival advantage in cancer may be the result of more complex interactions between sex hormones, genetic variability, including sex chromosomes and environment [8].

The influence of estrogens has been explored in sex-biased cancers, including MPM [9,10,11,12]. Estrogens are essential for human health and have broad biologic mechanisms of action such as regulating reproductive processes and the development and homeostasis of a wide range of tissues and organs, as well as playing a crucial role in the cardiovascular, nervous, immune systems, and bone metabolism [13]. In addition, tumor expression of hormone receptors plays an important role as an indicator of clinical prognosis of breast and genito-urinary cancers [14]. It has been reported that in patients with MPM, tumor expression levels of the estrogen receptor beta (ERβ) as assessed by immunohistochemistry (IHC) are correlated with survival [11]. Therefore, the elucidation of the molecular mechanisms associated with the survival advantage observed in women, in particular related to estrogen, may lead to prognostic and therapeutic advances for patients with MPM.

Over the last two decades, microarray-based technologies have led to a better understanding of tumor biology by identifying biomarkers for diagnosis, prognosis, or prediction of novel therapeutic strategies [15,16]. In the present study, transcriptomic analysis using gene expression microarrays was employed to identify estrogen-related genes that may be associated with observed differential prognosis between the two sexes. The RAS like estrogen regulated growth inhibitor (RERG) gene was identified as a female-specific prognostic biomarker.

## 2. Results

### 2.1. Identification of RERG

To identify genes associated with estrogen signaling potentially associated with longer survival in women with MPM, genes in the Estrogen Receptor Binding annotation of the Gene Ontology database (GO:ERB) were investigated using MPM datasets based on two microarray platforms (Codelink and Sentrix [17]). *RERG* was identified as the gene showing the highest variance in the Codelink microarray data and was the third-ranked gene in the Sentrix data (Table 1). *RERG* was found to be highly expressed across tumors in both cohorts.

**Figure 1:**
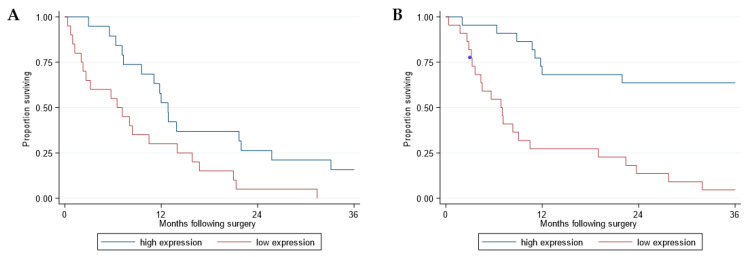
Survival analysis of the patients with malignant pleural mesothelioma (MPM) included in the Sentrix and Code-Link datasets according to *RERG* expression levels. Kaplan-Meier curves of overall survival according to *RERG* expression in the Sentrix (*n* = 39) (**A**) and CodeLink (*n* = 44) (**B**) datasets. Red and blue curves represent *RERG* expression lower and higher than the median levels, respectively. Overall survival was calculated from date of surgery. Survival curves were truncated at 36 months.

*RERG* expression (dichotomized at median expression levels for each dataset: Sentrix = 17.72; Codelink = 48.87) was analyzed in relation to survival. Overall survival (OS) was longer among patients with high *RERG* expression (above the median level) compared to patients whose tumors had low *RERG* expression in both Sentrix (median OS 12.8 versus 6.5 months, HR = 2.37, *p* = 0.014) and Codelink (median OS 40.7 versus 6.9 months, HR = 4.8, *p* < 0.001) datasets (Figure 1A,B, respectively).

### 2.2. Validation of Association of RERG Expression and Survival in a Selected Cohort of Epithelioid MPM Samples

**Figure 2:**
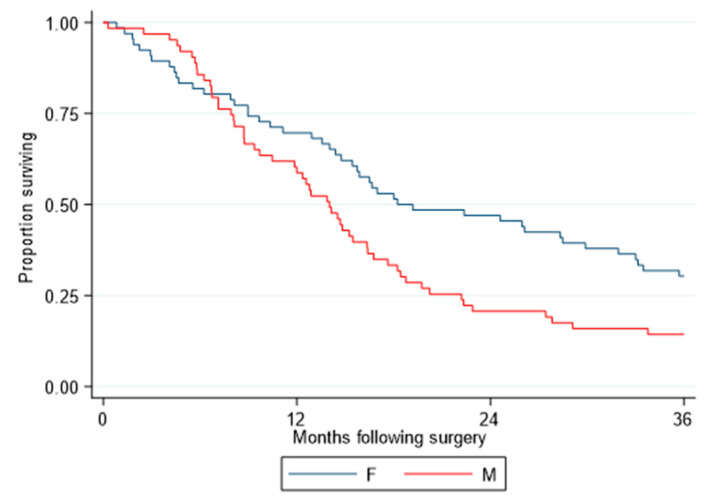
Survival analysis of the patients with MPM included in the selected cohort according to sex. Kaplan-Meier curves of overall survival between the two sexes in the selected datasets (*n* = 129). Red and blue curves represent male and female, respectively. Overall survival was calculated from date of surgery. Survival curves were truncated at 36 months.

**Figure 3:**
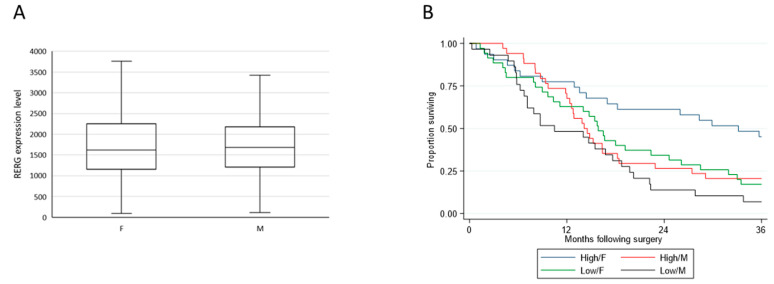
RERG analyses in relation to sex (**A**) and survival (**B**) in the Affymetrix cohort. Box plots (outliers not shown) illustrating *RERG* expression for female (*n* = 66) and male (*n* = 63) MPM patients (**A**). Kaplan-Meier curves of overall survival according to sex and *RERG* expression (*n* = 129) (**B**). Green and blue indicate low and high *RERG* expression in women, respectively, whereas black and red indicate low and high *RERG* expression in men. Overall survival was calculated from date of surgery. Survival curves were truncated at 36 months.

Using microarray data (Affymetrix, ThermoFisher, Carlsbad, CA, USA) from a selected cohort comprising only patients with epithelioid tumors resected by EPP, and with equal numbers of male and female cases matched by age and nodal status to control for the known prognostic covariates, OS remained statistically significantly different between the two sexes (*p* = 0.015), with the median survival for females being 18.2 months and that for males 14.0 months (HR = 1.64) (Figure 2). MPM samples displayed a range of *RERG* expression levels with men and women having similar distributions (*p* = 0.66) (Figure 3A). Cox regression revealed that longer OS was associated with high *RERG* expression in women (median 33.1 months, reference) compared to women with low *RERG* expression (15.9 months, HR = 2.05, *p* = 0.019) or to men with either low (10.5 months, HR = 3.04, *p* < 0.001) or high *RERG* expression (14.1 months, HR = 2.04, *p* = 0.022) (Figure 3B).

### 2.3. Biological Pathway Analyses

**Figure 4:**
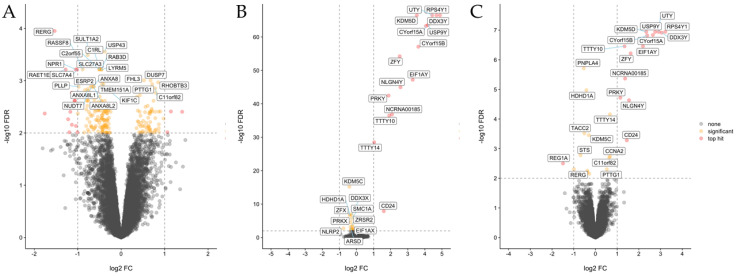
Differential expression analysis of 129 MPM tumors. Volcano plots representing differentially expressed genes between (**A**) tumors expressing high *RERG* vs. tumors exhibiting low *RERG* in women; (**B**) women and men; (**C**) tumors of women expressing high *RERG* vs. all the other samples. The −log10 false discovery rate (FDR) was plotted against log2 fold change (FC) values for all tested genes. The symbols on the negative and positive values of *x*-axis in each figure represent downregulated and upregulated genes, respectively. Symbols corresponding to significantly differentially expressed genes (FDR < 0.05) and top hit genes (FDR < 0.05 and 2-fold change) are colored in yellow and red, respectively.

To explore molecular features associated with sex and *RERG* expression, we searched for differentially expressed genes and enriched pathways using supervised analyses of the Affymetrix dataset. Among women, analysis of tumors expressing high levels of *RERG* vs. those with low levels identified differentially expressed genes related to integrin, extracellular matrix, and cell cycle pathways (Table S1; Figure 4A). By contrast, analyses of either women vs. men (Table S2; Figure 4B) or of women with high-*RERG*-expressing tumors vs. all others (Table S3; Figure 4C), the majority of differentially expressed genes mapped on the sex chromosomes.

### 2.4. Tumor Expression of Estrogen Receptors and Circulating Estradiol

Because two potential consensus ER binding sites had been identified within the 5′ upstream region of *RERG* gene [18], ESRα and ESRβ protein and gene expression were investigated in a cohort of MPM tumors representing a subset of the Affymetrix dataset (*n* = 95). Immunohistochemical analysis using ESRα antibody performed in this cohort was negative for ESRα protein expression in all the samples as previously reported [18]. Unfortunately, the antibodies used for previously published positive ERβ staining in mesothelioma [11] were not available to us to attempt to replicate that finding. Attempts to demonstrate ESRβ expression in MPM tumors with another commercially available antibody (H-150 sc-8974, Santa Cruz Biotechnology, Dallas, TX, USA) were unsuccessful.

The expression levels of the estrogen receptor genes were investigated in the Affymetrix microarray data to evaluate their potential prognostic role in the epithelioid sex-balanced cohort. These analyses revealed that levels of *ESR1* and *ESR2* expression were below the threshold for “reliable expression” (described in Methods), likely indicating absent or very low expression of these genes.

Measured estradiol levels in a subset of Affymetrix cohort cases with available plasma samples (25 male, 17 female) were not associated with OS in this cohort (HR = 0.84, *p* = 0.634).

## 3. Discussion

Sex-related differences in clinical characteristics of cancer have long been recognized. For example, some cancers induce higher mortality in men [19], whereas other tumors have shown significant differences in response to treatment in female patients [20]. The advent of high-throughput gene expression technologies has increased understanding of molecular correlates of malignancy, providing novel ways to stratify patients, determine prognosis, and predict sensitivity to therapeutic treatments (reviewed in [21]). Molecular signatures associated to cancer have demonstrated that some types of cancers have sex-biased gene expression [22].

In MPM, female sex has repeatedly been identified as a favorable prognostic factor ([5,6,23,24,25]. In a recent study including 18,799 patients with MPM [4], women were found independently associated with improved survival relative to men (*p* ≤ 0.001) [4]. However, only a few studies have investigated the role of the estrogen receptors or estrogen-regulated genes in relation to outcome [11,12]. *RERG* was identified among estrogen-related genes as having the highest variability in expression level in two sets of microarray data (an observation previously presented in abstract form [26]), with significant association to patient survival. In this study, this relationship was validated in a sex-balanced cohort controlled for other recognized prognostic factors. Interestingly, in this matched cohort, the levels of *RERG* expression were comparable between men and women. Nevertheless, OS following surgery-based multimodality therapy was found to be significantly longer in female patients whose tumors expressed high levels of *RERG,* whereas no significant association was observed in men.

*RERG* has been implicated in regulating cell proliferation in multiple cancers. *RERG* has been found frequently silenced by promoter CpG methylation in nasopharyngeal carcinoma, where it acts as a functional tumor suppressor by suppressing the extracellular signal‑regulated kinase (ERK)/NF-κB signaling pathway [27]. In addition, quantitative analysis of DNA methylation using real-time PCR in nasopharyngeal carcinoma samples showed that the methylation rates of *RERG* were significantly higher in primary tumor samples than in normal tissues [28]. In another study, ERK5 was able to promote prostatic carcinoma cell proliferation and migration by inhibiting RERG protein expression [29]. In breast cancer, high RERG protein expression was associated with longer disease-specific survival and distant metastasis-free interval independently of other prognostic variables [30]. Machine learning analyses of invasive breast carcinomas found that *RERG* was one of the highest ranked genes able to differentiate between ER+ luminal-like and ER- non-luminal breast cancers [30]. Microarray analysis on breast cancer HEK293 cell lines expressing or non-expressing ERβ identified *RERG* as one of the primary target genes of ERβ activation [31].

Surprisingly, despite demonstrating a statistically significant, sex-specific relationship between *RERG* expression and OS, no evidence of estrogen receptor expression was found at either the RNA or protein level in MPM tumor tissues, and no association between circulating estradiol levels and survival was observed. Although prior reports have demonstrated ESRβ immunostaining, and suggested a prognostic role of ESRβ expression in MPM [11,12,32], we were unable to detect evidence of estrogen receptor expression in MPM cells either by immunohistochemical staining using available antibodies, or using transcriptome data. The mechanism by which tumor *RERG* expression levels are related to patient outcome, specifically in women with MPM, remains unclear.

To discover novel molecular features associated with sex and *RERG* expression, we searched for differentially expressed genes and enriched pathways using supervised analyses of the matched validation dataset. Interestingly, one gene often found differently expressed was *DDX3Y*/*DDX3X*, mapping on the sex chromosome. *DDX3X* has been identified as frequently mutated in MPM [33]. High *DDX3X* expression has been also associated with aggressive phenotype in human malignancies [34]. Pathway analyses of women with high *RERG* expression vs. women with low *RERG* levels showed that several pathways associated with cell cycle and integrins were downregulated in women with high levels of *RERG*. Therefore, in women, MPM with high levels of *RERG* expression may reflect the epithelial part of the “histomolecular gradient” associated with epithelial-to-mesenchymal transformation across MPM tumors [35], and accordingly, with a more favorable clinical outcome.

This study has several limitations. First, *RERG* expression analyses have been restricted to transcriptome data. Available RERG antibodies were not successful on MPM tissues in demonstrating RERG protein activity. Protein levels do not always correspond with transcript levels because downstream controls on translation can have an impact on earlier transcriptional activity [36]. Second, the Affymetrix cohort necessarily involved selecting male cases matched to available females, who generally are younger and have epithelioid disease, introducing biases based on selection and age relative to unselected cohorts. Third, adjunctive treatment modalities may also introduce bias in survival analysis. However, no differences in application of such therapy were observed between male and female patients in the validation cohort. Similarly, RERG expression levels were not found to differ between women treated versus untreated with neoadjuvant chemotherapy, adjuvant chemotherapy, or adjuvant radiotherapy. Finally, this study failed to demonstrate expression of *ESR1* and *ESR2* or their corresponding proteins in MPM tumors. Additional investigations are needed to define the potential role of estrogen receptors in observed sex differences associated with MPM. Similarly, lack of evidence for a relationship between circulating estradiol levels and survival may be due to the limited number of subjects with available plasma samples for the estradiol analysis.

In this investigation, we have identified *RERG* as having novel relevance to MPM, in that *RERG* expression was identified as an independent prognostic biomarker among women with MPM. Further investigation is needed to clarify to what degree estrogens and/or estrogen receptors might play a role in MPM sex bias and by what mechanism higher *RERG* expression may contribute to the survival advantage observed for women with the disease.

## 4. Materials and Methods

The present study was a single-institution retrospective cohort analysis. All patients underwent surgical resection of their tumor at a single center by one of several thoracic surgeons experienced in mesothelioma surgery. Portions of their resected tumor specimens were stored frozen in an institutional biorepository.

### 4.1. Specimens and RNA Extraction

All tumor specimens were collected between 1992 and 2009 at surgery as discarded specimens from patients who provided informed consent, fresh frozen, stored, and annotated by the institutional tumor bank with the Institutional Review Board approval (Partners Protocol: 1999P001980; Dana Farber Cancer Institute Protocol No 98-063) at the Brigham and Women’s Hospital (Boston, MA, USA). RNA was extracted from tumor enriched (>80%) samples using the Trizol (ThermoFisher Scientific, Carlsbad, CA, USA) method in combination with RNeasy kit (Qiagen, Valencia, CA, USA). DNase (Qiagen) treatment was conducted per the manufacturer’s instructions. The RNA was quantified using an ND-1000 spectrophotometer (ThermoFisher Scientific), and its integrity (RIN ≥ 7) was determined using the Agilent 2100 Bioanalyzer (Agilent, Santa Clara, CA, USA).

### 4.2. Identification of Most Variable Estrogen Response Genes in MPM

Estrogen-related genes were ranked according to the variability in their expression levels [37]. The variance and the mean normalized expression for each gene in the Estrogen Receptor Binding annotation of the Gene Ontology database (GO:ERB) was calculated using a published dataset, Sentrix Human-6 Expression BeadChip (Illumina, San Diego, CA, USA) (39 MPM samples; 5 women and 34 men) [17] and previously unpublished gene expression profiles of a cohort of 44 MPM samples (16 women and 28 men) obtained with the GE Healthcare Codelink Human Whole Genome Bioarray. Tumor mRNAs were reversed transcribed into cDNA, labelled and hybridized to Codelink microarray probes using the CodeLink Expression Assay Reagent Kit (GE Healthcare-Amersham Biosciences, Chicago, IL, USA) per manufacturer’s instructions. Microarray chips were scanned using the GenePix 4000B instrument (Axon instruments, Union City, CA, USA). Array intensities were median-normalized using Codelink^TM^ Expression Analysis v4.1 software (GE Healthcare-Amersham Biosciences) to produce normalized intensity values for each gene probe. Codelink expression profile raw and normalized data are available at Gene Expression Omnibus (https://www.ncbi.nlm.nih.gov/geo/query/acc.cgi?acc=GSE163722)

Genes in Sentrix and CodeLink datasets were ranked by their relevant operationalization of variability. A log-linear model, either of the log2-transformed variance or as a function of log2-transformed mean normalized expression, was constructed to confirm that the observed high variance was not dependent on high gene expression. Residual values were calculated from the model and genes with more variability than predicted by mean expression were selected.

### 4.3. Microarray Validation and Functional Enrichment Analyses

In order to have sufficient power to explore sex differences in OS related to *RERG* expression, a third cohort of 132 MPM patients was selected to control for male predominance characteristic of unselected MPM cohorts, and for well-known prognostic factors following surgery including completeness of resection, sex, age, lymph node involvement and tumor histology. Equal numbers of specimens representing male and female patients were selected and matched based on lymph node status (N0: 20 (30%) female, 19 (30%) male; *p* = 1), and age (median (range): 57 (17–73) female, 58 (25–74) male *p* = 0.568). Each had complete tumor resection by extrapleural pneumonectomy and had pathological diagnosis of epithelioid MPM. Three samples were removed from the cohort due to poor quality of the extracted RNA. Microarray transcriptome data were obtained from the remaining 129 samples (66 women and 63 men) samples and linked to clinical and outcome data (Table 2).

Two hundred fifty ng of total RNA was amplified using the Ambion WT Expression Kit (ThermoFisher Scientific). The cRNA was hybridized to Affymetrix^®^ Human Gene 1.1 ST Array (ThermoFisher Scientific), labeled with GeneChip WT Terminal Labeling Kit (ThermoFisher Scientific), and then scanned with a GeneAtlas™ Workstation (ThermoFisher Scientific) as recommended by the manufacturer. Two additional samples (MAQCa and MAQCb) were added in the analysis for quality control across platforms [38]. In addition, two replicate samples were included to determine the variability of the expression values across the entire experiment. The arrays were normalized by RMA using Bioconductor.

*RERG*, *ESR1*, and *ESR2* gene expression levels are included in Table S4.

Expression profile raw data are available at Gene Expression Omnibus (https://www.ncbi.nlm.nih.gov/geo/query/acc.cgi?acc=GSE163722)

### 4.4. Gene Expression Analysis of Affymetrix Microarray Data

Differential expression analysis was performed with limma [39] using RMA normalized Affymetrix microarray expression data from the 129 epithelioid MPM samples. Probes with log2 expression above the log2 median expression (6.46 relative intensity) observed in negative control probes in at least 30 microarray samples were considered to be reliably expressed. Fifteen thousand one hundred and eighty-six probes with NCBI gene annotations and reliably expressed were tested for differential expression. Specifically, three differential expression comparisons between (1) tumors expressing high *RERG* vs. tumors exhibiting low *RERG* in women, (2) men and women; (3) tumors of women expressing high *RERG* vs. all the other samples, were performed using respective linear models and Empirical Bayes modulated t-statistics as described in the limma manual [40]. High RERG expression was defined as above the median expression observed in the cohort. False discovery rate (FDR) < 0.01 (for significance) [41] and absolute fold change >2 (to detect top hits) were used as thresholds to define differentially expressed genes in each comparison. Curated pathway gene sets were retrieved from the Molecular Signatures Database (MSigDB), and the fgsea package was used for gene set enrichment analysis (GSEA).

### 4.5. IHC and Estradiol Immunoassay

Paraffin-embedded tumor tissues were available for 95 out of 129 patients included in the Affymetrix cohort. IHC analysis of ESRα protein expression was performed using ESRα Antibody (RM-9101-S1 clone SP1, Neomarkers, Portsmouth, NH, USA; 1:40 dilution) and the Leica Biosystems Refine Detection Kit with citrate antigen retrieval on the Leica Bond III automated staining platform. Fallopian tube tissue was used as positive control. A U.S. board-certified pulmonary pathologist (LCR) reviewed all IHC results and provided interpretation. Because the antibody (H-150 sc-8974, Santa Cruz Biotechnology; dilution 1:100) failed to detect ERβ in two MPM samples and the transcriptome data showed expression of ERS2 below detection limits in most cases, further testing of ERβ antibodies was not pursued. Human plasma was available from 42 patients included 17 from females and 25 from males in the Affymetrix cohort. The levels of estradiol in human plasma were determined using the Access Chemiluminescent Immunoassay for estradiol (Beckman Coulter, Brea, CA, USA) at the GCRC Core Laboratory (Brigham and Women’s Hospital, Boston, WA, USA).

### 4.6. Statistical Analysis

Overall survival (OS) was defined from the date of definitive surgery until the date of death or was censored at the date of last follow-up for patients who had not died at their latest contact. The Kaplan-Meier method was used to estimate the OS functions, with group differences expressed as a hazard ratio that was estimated by the proportional hazards model. The normalized expression levels of *RERG*, *ESR1*, and *ESR2* were arranged from low to high expression, and the samples were divided initially into quartiles for exploratory survival analysis. Within patient subgroups, adjacent quartiles with similar survival were pooled for further analysis of survival differences. Wilcoxon rank-sum test was used to compare the distribution of *RERG* expression levels and estradiol levels between sexes and histological types. All *p*-values were based on a two-sided hypothesis. Data analysis was performed using SAS 9.4 (SAS Institute, Cary, NC, USA) and Stata (College Station, TX, USA; Version 15.1).

## 5. Conclusions

Because many cancers exhibit significant differences in incidence, prognosis, and response to treatment between men and women, and MPM exhibits a strong sex bias and a survival advantage for women patients, this study attempted to identify estrogen-related biomarkers that might be detectable in tumor biopsies. *RERG* expression was shown to be an independent predictor of prognosis in women when controlling for other known prognostic factors, suggesting that *RERG* may play a role in the survival advantage observed among women with MPM. However, because neither protein nor gene expression of ERα and ERβ were detectable in this cohort, and levels of circulating estradiol were not found to be related to OS, additional analysis is needed to determine the role of *RERG* in MPM and its potential relationship with estrogen.

## Figures and Tables

**Table 1:** The 10 most variable genes in the Estrogen Receptor Binding annotation of the Gene Ontology database using Codelink and Sentrix microarray data.

ProbeID	Gene	Mean	Variance	CV^2 *	Microarray
GE87411	*RERG*	45.00	832.24	0.41	Codelink
GE81045	*PARP1*	101.99	517.95	0.05	Codelink
GE56173	*LEF1*	11.21	156.55	1.25	Codelink
GE88499	*FUS*	2.13	83.90	18.42	Codelink
GE80030	*ACTN4*	17.44	68.11	0.22	Codelink
GE80030	*FUS*	17.44	68.11	0.22	Codelink
GE818783	*TRIP4*	26.36	53.05	0.08	Codelink
GE62204	*NKX3-1*	11.50	49.68	0.38	Codelink
GE695752	*RERG*	7.47	39.00	0.70	Codelink
GE54705	*SRC*	23.38	29.39	0.05	Codelink
ILMN_20678	*TACC1*	63.80	508.09	0.12	Sentrix
ILMN_12963	*WBP2*	70.78	412.02	0.08	Sentrix
ILMN_12434	*RERG*	20.61	248.54	0.59	Sentrix
ILMN_3056	*PARP1*	40.17	202.99	0.13	Sentrix
ILMN_2259	*CNOT1*	28.51	93.19	0.11	Sentrix
ILMN_15515	*PHB2*	19.11	45.92	0.13	Sentrix
ILMN_137682	*CTNNB1*	12.80	29.96	0.18	Sentrix
ILMN_11329	*TAF10*	22.98	27.66	0.05	Sentrix
ILMN_4339	*NRIP1*	13.43	19.14	0.11	Sentrix
ILMN_20599	*NCOA6*	16.16	15.07	0.06	Sentrix

* CV^2 = coefficient of variation squared.

**Table 2:** Clinical and histopathologic characteristics of epithelioid cohort.

	Sentrix	CodeLink	Affymetrix
**Evaluable for analysis**	39	44	129
Alive at last follow-up	0	1	12
**Sample Collection (years)**	2002–2006	2001–2006	1992–2009
**Age at surgery, years**			
Median (range)	62 (38–75)	61 (37–80)	57 (17–74)
**Sex**			
Male	34	28	63
Female	5	16	66
**Histologic subtype**			
Epithelioid	25	23	129
Biphasic	8	19	
Sarcomatoid/Desmoplastic	6	2	
**Nodal Stage**			
N0	19	16	39
N1–N3	16	21	90
Nx	4	7	
**Self-reported asbestos exposure**			
Yes	24	24	76
No	11	11	43
Unknown	4	9	10
**Neoadjuvant Chemotherapy**			
Yes	6	5	16
No	33	39	113
**Adjuvant Chemotherapy**			
Yes	18	24	56
No	18	17	57
Unknown	3	3	16
**Adjuvant Radiation Therapy**			
Yes	14	18	60
No	22	23	52
Unknown	3	3	17
**Surgical procedure**			
Extrapleural Pneumonectomy	39	31	129
Pleurectomy/Decortication		6	
Extended Pleurectomy/Decortication		3	
Partial Pleurectomy/Decortication		4	

## Data Availability

The data presented in this study are openly available at Gene Expression Omnibus (Accession #: GSE42977, GSE163722).

## Supplementary Materials

The following are available online at https://www.mdpi.com/2072-6694/13/3/565/s1, Table S1: List of the differentially-expressed genes (A) and pathways (B) in tumors expressing high levels of RERG vs. tumors expressing low levels of RERG in women, Table S2: List of the differentially-expressed genes (A) and pathways (B) in women vs. men, Table S3: List of the differentially-expressed genes (A) and pathways (B) in tumors expressing high levels of RERG in women vs. all the other samples included in the analysis, Table S4: RERG, ESR1 and ESR2 gene expression levels in the Affymetrix dataset.
